# Early Complete Molecular Response to First-Line Nilotinib in Two Patients with Chronic Myeloid Leukemia Carrying the p230 Transcript

**DOI:** 10.1155/2013/871476

**Published:** 2013-07-11

**Authors:** Marianna Greco, Giovanni Caocci, Giorgio La Nasa

**Affiliations:** ^1^Hematology Unit and Bone Marrow Transplantation Center, “R. Binaghi” Hospital, Via Is Guadazzonis 3, 09126 Cagliari, Italy; ^2^Hematology, Department of Medical Sciences, University of Cagliari, 09100 Cagliari, Italy

## Abstract

Chronic myeloid leukemia (CML) with the rare fusion gene e19a2, encoding a p230 protein, has been described in patients with typical or rather aggressive clinical course. Although tyrosine kinase inhibitors (TKIs) induce a substantial cytogenetic and molecular response in all phases of CML, a minority of p230 positive patients have been treated with TKIs. We report two cases of CML patients carrying the p230 transcript, who achieved fast and deep complete molecular response (CMR) after frontline treatment with nilotinib. Our results suggest the use of nilotinib as frontline agent for the treatment of this CML variant.

## 1. Introduction

The molecular basis of chronic myeloid leukemia (CML) is the BCR/ABL fusion gene, which originates from a balanced translocation between the long arms of chromosomes 9 and 22, t(9;22)(q34;q11), leading to the Philadelphia (Ph) chromosome formation. According to the different BCR gene exons involved, it is possible to recognize the transcript b2a2 or b3a2, coding for a p210 protein, e1a2 which codes for a p190 protein, and the less common fusion gene e19a2, encoding a p230 protein. The p230 was first identified in patients showing a mild form of CML, designated as Ph+ chronic neutrophilic leukaemia (CNL), a rare disorder characterized by moderate and persistent neutrophilia without precursors on peripheral blood smear, absent or normal splenomegaly, and a benign clinical course [1, 2]. Subsequently, the e19a2 rearrangement was detected in patients with typical CML or in those with a rather aggressive clinical course [3–5]. Although tyrosine kinase inhibitors (TKIs) induce a substantial cytogenetic and molecular response in all phases of CML, a minority of these patients have been treated with TKIs [6–9].

## 2. Case Presentation

We report two cases of CML patients carrying the p230 transcript, who achieved fast and deep complete molecular response (CMR), defined as the absence of detectable BCR-ABL transcripts by RQ-PCR, after frontline treatment with nilotinib.

The first patient was a 40-year-old male diagnosed with chronic phase CML in October 2010. The examination of his peripheral blood showed a hemoglobin (Hb) level of 13.2 g/dL, white blood cell (WBC) count of 59 × 10^9^/L, and a platelet count of 236 × 10^9^/L. No organomegaly was observed. After bone marrow aspirate, the cytogenetic analysis revealed 46, XY, t(9;22)(q34;q11.2) karyotype in 100% of metaphases. Qualitative PCR detected the sole presence of the e19a2 transcript. According to Sokal score, the patient was classified as intermediate-1 risk class. He was started on nilotinib 600 mg per day. After 3 months, he achieved complete cytogenetic response (CCyR) and CMR, confirmed by nested rt-PCR (Kits from Nanogen Advanced Diagnostics, Turin, Italy). Currently, 31 months after the start of the treatment, the patient continues to take 600 mg nilotinib per day; he feels well and CMR is confirmed. 

The second patient, a 41-year-old woman, was diagnosed with chronic phase CML in November 2010. Her blood examination showed the following hematological parameters: Hb 12.5 g/dL, WBC 60 × 10^9^/L, and platelets 1796 × 10^9^/L. The classic Ph karyotype was confirmed in all the metaphases. RT-PCR detected the presence of the e19a2 transcript. After starting frontline treatment with nilotinib 600 mg per day, the patient achieved CCyR and CMR in 6 and 8 months, respectively. Currently, after 30 months, she is well and in CMR.

## 3. Discussion

There are only few reports about the treatment with imatinib and only one reporting data on nilotinib treatment in patients with the e19a2 rearrangement, with varying clinical outcomes [6–9]. So far, the only patient treated with first-line nilotinib achieved a favorable molecular response, despite several treatment interruptions and dose reductions [9]. Interestingly, the use of nilotinib has induced a fast CMR also in our two patients who maintained a complete molecular response for over three years until today. Analysis of killer immunoglobulin-like receptors (KIRs) of our patients showed a decrease in the frequency of the KIR2DL2 inhibitory gene, homozygosity for KIR haplotype A, and lower number of inhibitory KIR genes, which have been suggested to represent possible predictors of CMR [10]. The present paper recommends the use of nilotinib as frontline agent for the treatment of this CML variant, according to the evidence of a deep and rapid molecular response obtained in our patients. This finding could help clinicians to decide in the future whether to stop TKI treatment in patients with the e19a2 rearrangement.
